# When the Beat Breaks the Heart: A Case of Arrhythmia-Induced Mid-ventricular Takotsubo Cardiomyopathy

**DOI:** 10.7759/cureus.108786

**Published:** 2026-05-13

**Authors:** Rana Kashif Riaz, Rivya Mathews, Abdul Maher

**Affiliations:** 1 Cardiology, Midland Metropolitan University Hospital, Birmingham, GBR

**Keywords:** arrhythmia, case report, mid-ventricular variant, stress-induced cardiomyopathy, takotsubo cardiomyopathy, ventricular tachycardia

## Abstract

Takotsubo cardiomyopathy (TTC) is a transient and reversible form of left ventricular systolic dysfunction that often mimics acute coronary syndrome and is usually precipitated by emotional or physical stress. We describe an unusual presentation of mid-ventricular TTC in which ventricular arrhythmia was the dominant clinical feature and a probable precipitating factor. A 71-year-old woman presented with left shoulder pain and palpitations in the absence of chest pain or an identifiable emotional stressor. Initial electrocardiography demonstrated frequent runs of rapid non-sustained ventricular tachycardia, with a markedly elevated troponin I level of 2188 ng/L. Coronary angiography showed unobstructed epicardial coronary arteries with delayed contrast clearance. Cardiac magnetic resonance imaging revealed severe left ventricular systolic dysfunction (left ventricular ejection fraction (LVEF) 20%-25%) with isolated mid-ventricular akinesia and preserved basal and apical contractility, consistent with a mid-ventricular variant of TTC. Management focused on arrhythmia control and supportive heart failure therapy, with acute stabilisation using intravenous amiodarone followed by beta-blockade with bisoprolol and guideline-directed medical therapy. The ventricular arrhythmia resolved, and left ventricular systolic function improved significantly, with follow-up cardiac magnetic resonance imaging demonstrating recovery of LVEF to 50%. Device therapy was not pursued, given the reversible nature of TTC. This case highlights ventricular arrhythmia as both a presenting manifestation and a possible trigger of TTC and emphasises the importance of considering atypical variants in patients presenting with unexplained cardiomyopathy and ventricular arrhythmias. Multimodality imaging remains essential for accurate diagnosis and for guiding appropriate management in such cases.

## Introduction

First described in Japan in 1990, Takotsubo cardiomyopathy (TTC) is an acute, reversible cardiac syndrome characterised by transient regional wall motion abnormalities that mimic an acute coronary syndrome [1]. It predominantly affects postmenopausal women and is frequently preceded by a significant emotional or physical stressor, leading to its common name, "broken-heart syndrome" [2]. The prevailing pathophysiological hypothesis centres on catecholamine-mediated myocardial stunning and microvascular dysfunction [3].

While apical ballooning is the classic form, several atypical variants, including mid-ventricular, basal (inverted), and focal, are increasingly recognised [4]. Diagnosis relies on established 2018 International Expert Consensus (InterTAK) criteria, including characteristic regional wall motion abnormalities extending beyond a single coronary territory and absence of obstructive coronary artery disease [5].

We report an atypical case of mid-ventricular TTC that presented with ventricular tachyarrhythmia as the dominant feature in the absence of any identifiable stress trigger. This case highlights a possible association between the arrhythmia and myocardial dysfunction, where both conditions appear closely linked, underscoring the diagnostic challenge and the need for an integrated management approach.

## Case presentation

A 71-year-old woman with a background of hypertension and hypercholesterolaemia presented to the emergency department with a one-day history of left shoulder pain radiating to her back, neck, and jaw. She attributed the pain to a chronic musculoskeletal condition but reported new-onset palpitations associated with intermittent dizziness. She explicitly denied chest pain, emotional distress, or recent infectious symptoms. Her family history was notable for the sudden, unexplained death of a brother during sleep at the age of 44 years.

On arrival, she was tachycardic, with heart rates fluctuating between 144 and 185 beats per minute, accompanied by borderline haemodynamic status, with an admission blood pressure of 111/92 mmHg and systolic blood pressure fluctuating between 90 and 100 mmHg during arrhythmic episodes. Electrocardiography demonstrated frequent runs of non-sustained ventricular tachycardia (Figure 1). The remainder of the physical examination was unremarkable. High-sensitivity troponin I was markedly elevated at 2188 ng/L (reference range <5 ng/L). Additional laboratory parameters are summarised in Table 1. She was treated acutely with a 300 mg intravenous bolus of amiodarone as first-line therapy due to its established efficacy in ventricular arrhythmias and relatively favourable haemodynamic profile compared with other agents.

**Figure 1:**
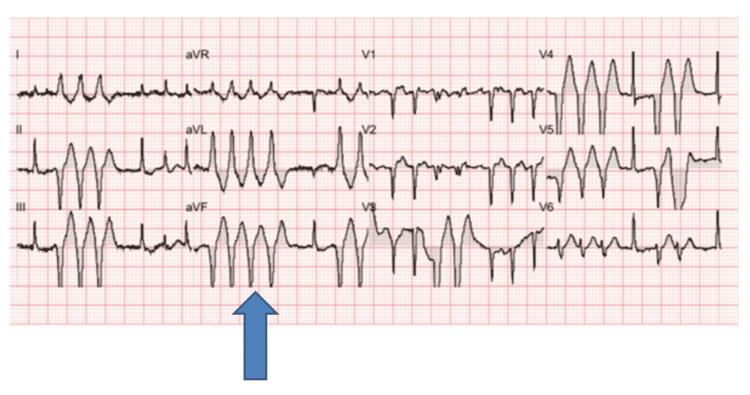
Electrocardiogram strip demonstrating a run of non-sustained ventricular tachycardia on admission.

**Table 1:** Patient's blood test results on Days 1 and 3 of admission with normal reference ranges

Parameter	Patient’s result on Day 1 of admission	Patient’s result on Day 3 of admission	Normal reference range
White cell count	8.3 x10^9^/L	12 x10^9^/L	4-10 x10^9^/L
Neutrophil count	5.6 x10^9^/L	9.8 x10^9^/L	2-7 x10^9^/L
Sodium	135 mmol/L	122 mmol/L	133-146mmol/L
Potassium	4.5 mmol/L	5 mmol/L	3.5-5.3 mmol/L
Estimated glomerular filtration rate (eGFR)	62 mL/min/1.73m^2^	90 mL/min/1.73m^2^	>90 mL/min/1.73m^2^
C-reactive protein (CRP)	1 mg/L	37 mg/L	0-10 mg/L
Troponin I	2188.4 ng/L	-	<5 ng/L
Alanine transaminase (ALT)	58 IU/L	313 IU/L	0-55 IU/L

On review of the presenting electrocardiogram, the arrhythmia consisted of frequent runs of non-sustained ventricular tachycardia with a monomorphic morphology, demonstrating broad QRS complexes and a consistent pattern across episodes, suggesting a single ventricular focus rather than a polymorphic arrhythmia. The episodes were short, self-terminating, and recurrent, without progression to sustained ventricular tachycardia or haemodynamic instability.

Although a formal quantitative assessment of arrhythmic burden (e.g., percentage of ventricular ectopy or total nonsustained ventricular tachycardia (NSVT) episodes over time) was not available, continuous monitoring confirmed frequent recurrent NSVT during the acute phase, which resolved following antiarrhythmic therapy and clinical stabilisation.

In the context of acute severe but reversible left ventricular systolic dysfunction, absence of prior arrhythmic history, and subsequent recovery of ventricular function, the arrhythmia was considered more likely secondary to transient myocardial dysfunction rather than a primary electrical disorder.

Diagnostic assessment

Coronary angiography demonstrated unobstructed epicardial coronary arteries with delayed contrast clearance (Figure 2), a finding suggestive of coronary microvascular dysfunction. A point-of-care transthoracic echocardiogram was limited by ongoing tachyarrhythmia but suggested severe left ventricular systolic impairment. As such, the working diagnosis was of ventricular tachyarrhythmias in the context of a non-ischaemic non-dilated cardiomyopathy.

**Figure 2:**
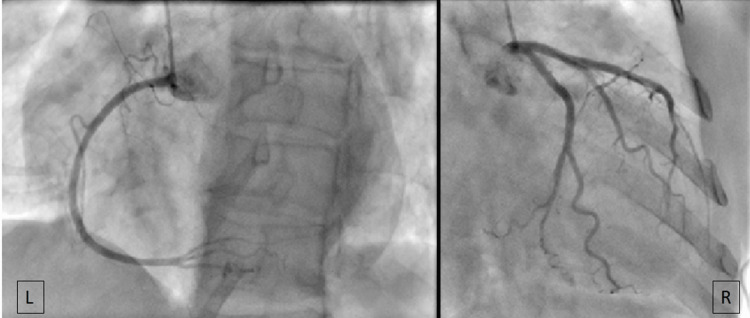
Coronary angiography demonstrated unobstructed LAD with delayed contrast clearance: (L) LAO of RCA, (R) RAO caudal view LAD: left anterior descending artery; LAO: left anterior oblique; RCA: right coronary artery; RAO: right anterior oblique

Following coronary angiography, the patient developed hypotension with a blood pressure of 98/58 mmHg, accompanied by recurrent episodes of NSVT, necessitating initiation of a continuous intravenous amiodarone infusion. The amiodarone infusion was not continued long-term; it was used as a time-limited intervention with close haemodynamic monitoring. Blood pressure remained stable within the aforementioned range without requiring vasopressor support. Alternative agents, including lidocaine, were considered; however, amiodarone was preferred initially due to its broader antiarrhythmic efficacy, particularly in the context of uncertain underlying aetiology and reduced left ventricular systolic function.

A subsequent comprehensive transthoracic echocardiogram demonstrated a non-dilated left ventricle with severely impaired systolic function, with a visually estimated left ventricular ejection fraction (LVEF) of 20%-25%. Regional wall motion assessment revealed akinesia of all mid-ventricular segments with preserved basal contractility, consistent with a mid-ventricular pattern of involvement (Figures 3-5).

**Figure 3:**
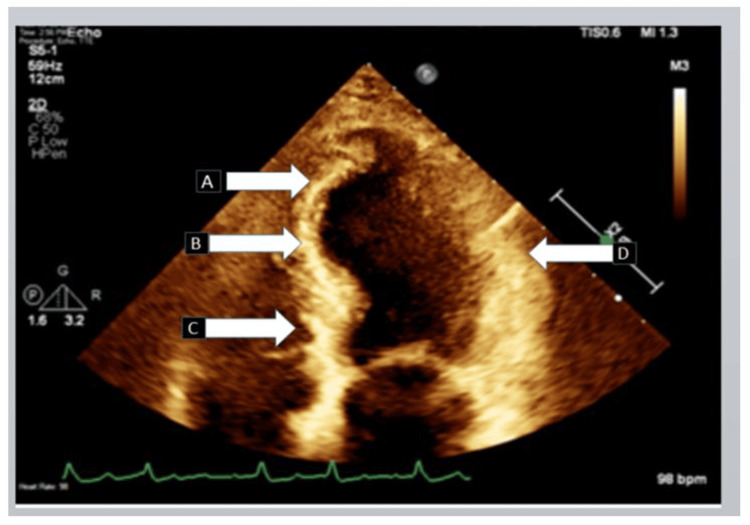
Echocardiogram (apical four-chamber view, end-systole) showing preserved apical septal contractility: (A) akinesia of the mid inferoseptal, (B) basal inferoseptal hypercontractility, (C) and mid anterolateral segments akinesia (D).

**Figure 4:**
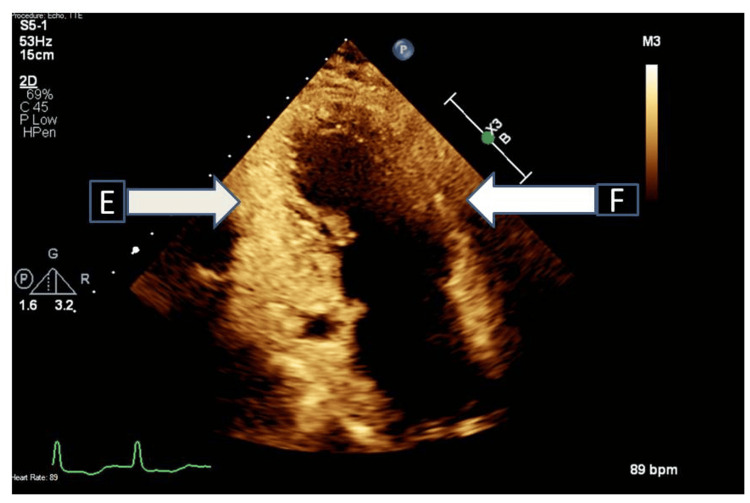
Echocardiogram (apical two-chamber view) showing akinesia of the mid anterior (F) and mid inferior (E) segments.

**Figure 5:**
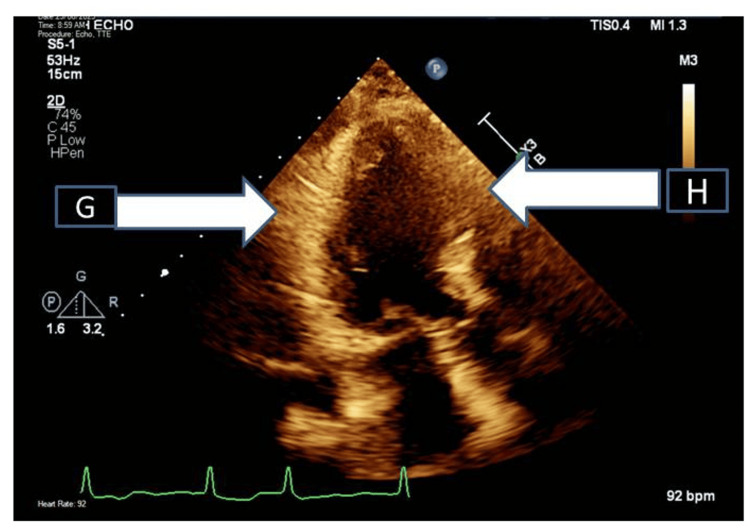
Echocardiogram (apical three-chamber view) showing akinesia of mid-inferolateral (G) and mid-anteroseptal (H) segments.

Cardiac magnetic resonance imaging confirmed the diagnosis, demonstrating akinesia of the mid-ventricular segments with preserved basal and apical motion, consistent with the mid-ventricular variant of TTC. Left ventricular systolic function had improved, with an estimated LVEF of 50%. Late gadolinium enhancement showed subtle mid-wall septal fibrosis without evidence of myocardial infarction or myocarditis. The left atrium was moderately dilated (Figure 6).

**Figure 6:**
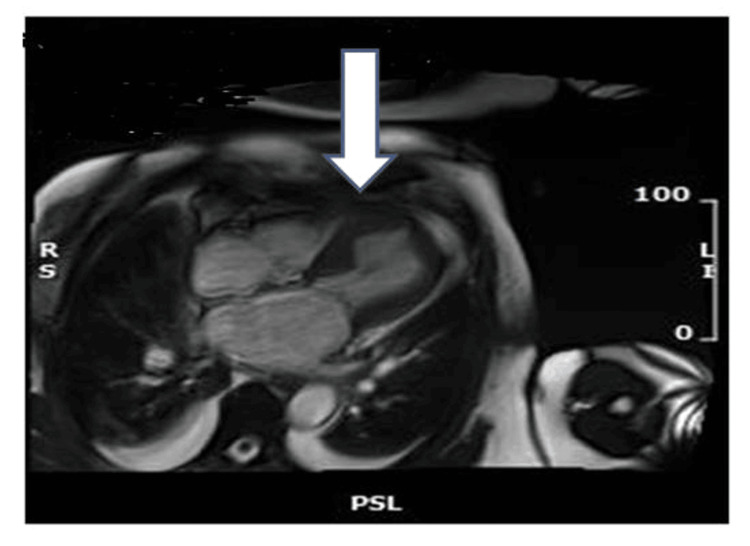
Cardiac magnetic resonance (axial four-chamber cine sequence) showing mid-ventricular akinesia with preserved basal and apical contraction (white arrows).

The left shoulder pain was initially attributed to a musculoskeletal cause, as described by the patient. However, given the elevated troponin and subsequent diagnosis, it may also represent an atypical presentation of myocardial injury, which is recognised in TTC.

During hospitalisation, the patient developed transient hyponatraemia and elevated liver transaminases, with a peak alanine aminotransferase level of 313 IU/L. These abnormalities were attributed to congestive hepatopathy and a possible contribution from amiodarone therapy and resolved with supportive management.

Therapeutic intervention and clinical course

Management focused on two parallel objectives: suppression of ventricular arrhythmia and support of left ventricular recovery.

Arrhythmia Control

Following initial stabilisation with intravenous and oral amiodarone, the medication was discontinued after review by the electrophysiology team. Long-term rate control and prevention of adverse remodelling were achieved with bisoprolol, which was titrated to 3.75 mg twice daily.

Heart Failure Management

Guideline-directed medical therapy was initiated, including dapagliflozin, ramipril, and spironolactone. Spironolactone was temporarily withheld due to hyperkalaemia and later reintroduced.

Additionally, the patient exhibited borderline systolic blood pressure (90-100 mmHg) during the acute phase, which limited the safe initiation of angiotensin receptor-neprilysin inhibitors (ARNI), given their known vasodilatory effects and potential to exacerbate hypotension.

Instead, guideline-directed medical therapy was initiated with a low-dose angiotensin-converting enzyme inhibitor (ramipril) alongside other supportive measures. This approach allowed for cautious optimisation of therapy while monitoring haemodynamic stability and assessing for recovery of ventricular function. As the patient demonstrated significant improvement in LVEF (to 50%), the indication for escalation to ARNI therapy was no longer present.

Device Consideration

After a multidisciplinary electrophysiology discussion, implantable cardioverter-defibrillator implantation was not recommended, given the anticipated reversibility of myocardial dysfunction.

The patient’s clinical condition stabilised with complete resolution of ventricular arrhythmias and significant symptomatic improvement. She was discharged on day ten in sinus rhythm on optimised medical therapy.

Follow-up and outcomes

A summary of the clinical course, key investigations, and management decisions is provided in Table 2.

**Table 2:** Timeline of key clinical events, diagnostic findings, and interventions. ACEi: angiotensin-converting enzyme inhibitor; ECG: electrocardiogram; EP: electrophysiology; IV: intravenous; LFTs: liver function tests; LVEF: left ventricular ejection fraction; MDT: multidisciplinary team; MRA: mineralocorticoid receptor antagonist; MRI: magnetic resonance imaging; NSVT: nonsustained ventricular tachycardia; TTC: Takotsubo cardiomyopathy.

Date	Clinical events and findings	Interventions and management decisions
Day 1	Presentation with shoulder pain, palpitations, and NSVT on ECG. Troponin I = 2188 ng/L.	IV amiodarone bolus. Coronary angiography (normal arteries, slow flow was diffuse across all epicardial vessels).
Day 1 (post angio)	Hypotension, recurrent NSVT.	IV amiodarone infusion initiated.
Day 2	Formal echo: LVEF = 20-25% with mid-ventricular akinesia.	Start oral amiodarone, bisoprolol, dapagliflozin, spironolactone, ramipril.
Day 3	Hyponatraemia; deranged LFTs. First EP MDT review.	ACEi/MRA held; amiodarone stopped; bisoprolol dose increased.
Day 6	Cardiac MRI confirmed mid-ventricular TTC with subtle fibrosis; LVEF = 50%.	Low-dose spironolactone re-initiated.
Day 10	Clinically stable. Second EP MDT review.	Discharged on bisoprolol, dapagliflozin, ramipril, and spironolactone. Follow-up planned.
3 months	Follow-up	Follow-up cardiac MRI and seven-day ambulatory electrocardiographic monitoring

At the time of discharge, the patient was asymptomatic and in sinus rhythm. A structured follow-up plan was arranged, including repeat cardiac magnetic resonance imaging and seven-day ambulatory electrocardiographic monitoring at three months to assess sustained recovery of left ventricular function and to evaluate for recurrent ventricular arrhythmia or the need for further intervention.

## Discussion

This atypical case highlights the potential relationship between ventricular arrhythmia and TTC. In this patient, ventricular tachyarrhythmia was the dominant presenting feature of a mid-ventricular variant of TTC, an atypical pattern characterised by isolated mid-ventricular akinesia with preserved basal and apical function [4]. The presentation occurred in the absence of an identifiable emotional or physical stressor, creating a diagnostic challenge.

The 2018 InterTAK diagnostic criteria for Takotsubo syndrome define the condition by the presence of (1) transient left ventricular systolic dysfunction, manifesting as hypokinesia, akinesia, or dyskinesia, with regional wall motion abnormalities that typically extend beyond a single epicardial coronary artery distribution, including apical, mid-ventricular, basal, or focal patterns, and with possible right ventricular involvement; (2) the occurrence of an emotional, physical, or combined trigger, although this is not obligatory; (3) the presence of new electrocardiographic abnormalities, such as ST-segment elevation, ST-segment depression, T-wave inversion, or QTc prolongation; (4) modest elevation in cardiac biomarkers (e.g., troponin and creatine kinase) with a disproportionate rise in natriuretic peptides; (5) the absence of evidence for infectious myocarditis; (6) recognition that significant coronary artery disease may coexist and does not exclude the diagnosis; and (7) a predominance in postmenopausal women, although the condition can occur in both sexes and across age groups [5]. In addition, the consensus introduced the InterTAK diagnostic score, incorporating clinical variables, including female sex, emotional or physical triggers, absence of ST-segment depression, presence of psychiatric or neurologic disorders, and QTc prolongation, to estimate the probability of TTS and aid differentiation from acute coronary syndromes [5].

Cardiac magnetic resonance imaging was central to confirming the diagnosis and informing the prognosis. The pattern of isolated mid-ventricular akinesia with preserved basal and apical function is characteristic of this variant of TTC [3]. The absence of myocardial infarction or myocarditis further supported the diagnosis of a reversible cardiomyopathy. As previously outlined, these findings are consistent with established diagnostic criteria for TTC, which emphasise transient regional wall motion abnormalities in the absence of obstructive coronary artery disease and alternative causes of myocardial injury [6].

The key diagnostic issue was determining the direction of causality. Ventricular arrhythmias have been reported in approximately 8% of TTC cases and are most often described as secondary complications arising during the acute phase of myocardial dysfunction [7].

Ventricular tachyarrhythmia may represent a possible precipitating factor in this case; however, a causal relationship cannot be definitively established. The temporal association between arrhythmia resolution and subsequent improvement in left ventricular function suggests a potential interaction, although arrhythmia may also occur as a consequence of underlying myocardial dysfunction (Figure 7) [9]. This supports the presence of a self-perpetuating cycle in which ventricular arrhythmia precipitates myocardial dysfunction, which in turn increases susceptibility to further electrical instability. 

**Figure 7:**
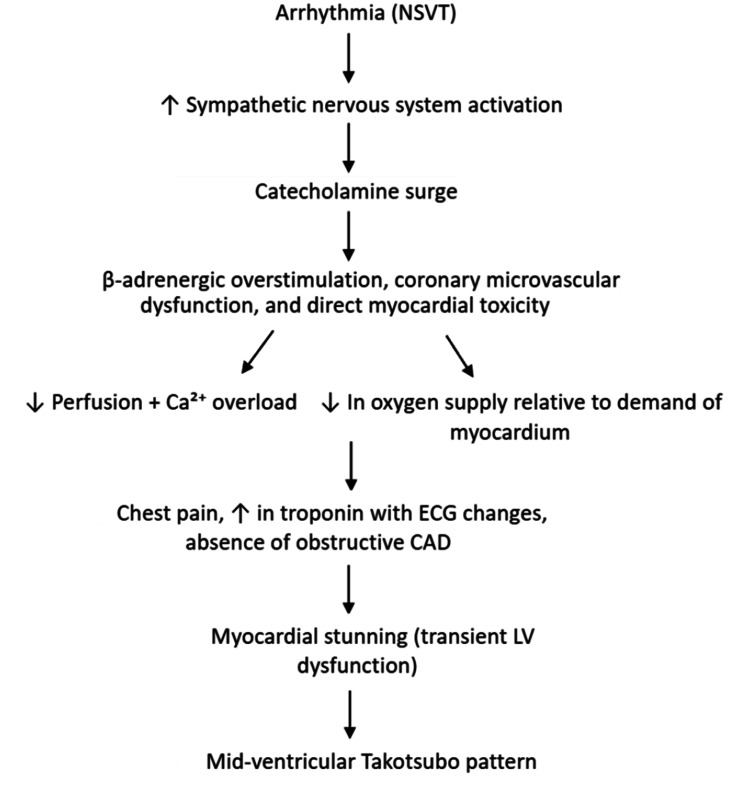
llustrative schematic showing the proposed mechanism of catecholamine excess leading to myocardial stunning in TTC TTC: Takotsubo cardiomyopathy; NSVT: nonsustained ventricular tachycardia; Ca2+: calcium; LV: left ventricular; CAD: coronary artery disease This figure has been created by the authors using Microsoft PowerPoint (Microsoft Corp., Armonk, NY, USA) and adapted from [9].

While tachyarrhythmia may theoretically contribute to sympathetic activation and myocardial injury, this relationship remains speculative in this case and should be interpreted with caution. This differs from tachycardia-induced cardiomyopathy, where sustained arrhythmia is the primary driver of ventricular dysfunction.

The presence of subtle mid-wall fibrosis, although not a classical feature, has been described in selected cases and may represent residual myocardial injury following catecholamine-mediated stress or, alternatively, reflect pre-existing subclinical myocardial fibrosis, potentially related to age or comorbidities [8]. Importantly, such fibrosis may act as a substrate for ventricular arrhythmia, which has implications for risk assessment and follow-up.

Management required a dual-focused approach addressing both electrical instability and transient myocardial dysfunction. Acute suppression of ventricular arrhythmia with amiodarone was necessary to stabilise the patient and interrupt the cycle of electrical-mechanical impairment. Subsequent beta-blocker therapy provided longer-term rate control, attenuation of catecholamine-mediated injury, and protection against recurrent arrhythmia. Deferral of implantable cardioverter-defibrillator implantation was appropriate, given the reversible nature of TTC and evidence supporting favourable outcomes with medical therapy alone [9]. 

This case is consistent with emerging literature demonstrating that ventricular arrhythmias, particularly ventricular tachycardia, may represent a significant and occasionally presenting manifestation of TTC [10-12]. In the absence of a fixed myocardial scar, proposed mechanisms include triggered activity and transient conduction heterogeneity related to catecholamine excess. Clinicians should therefore maintain a high index of suspicion for TTC in patients presenting with unexplained cardiomyopathy and ventricular arrhythmias, particularly in postmenopausal women, even when a classical stressor is not apparent.

Although apical ballooning is the most frequently recognised pattern, atypical variants including mid-ventricular, basal (inverted), and focal forms are increasingly reported. Diagnosis is based on established criteria that include transient wall motion abnormalities extending beyond a single epicardial coronary territory, the absence of obstructive coronary artery disease, supportive electrocardiographic changes or biomarker elevation, and exclusion of alternative causes such as myocarditis or pheochromocytoma [6].

Ventricular arrhythmias are recognised complications of TTC, but are most often described as secondary phenomena occurring in the setting of acute myocardial dysfunction. Presentation with ventricular tachyarrhythmia as the dominant initial feature, particularly in the absence of an identifiable stress trigger, remains uncommon and is less well characterised in the literature. We report a case of mid-ventricular TTC in which ventricular tachyarrhythmia was the primary presenting manifestation and a plausible precipitating factor. This case adds to the existing literature by highlighting a potential association between electrical instability and transient myocardial dysfunction and underscores the diagnostic and management challenges posed by atypical presentations of TTC.

## Conclusions

TTC may present with ventricular arrhythmia as the primary or sole initial manifestation, even in the absence of an identifiable emotional or physical stressor. While ventricular arrhythmias are recognised complications of TTC, they are most commonly described as secondary phenomena during the acute phase of myocardial dysfunction. Multimodality imaging, including coronary angiography and cardiac magnetic resonance imaging, is essential for accurate diagnosis, exclusion of alternative causes, and guidance of management in atypical presentations of TTC. Management of atypical cases requires a multidisciplinary approach, with close collaboration between heart failure and electrophysiology teams to address both electrical instability and reversible ventricular dysfunction.
